# Design and methods of the Hospital Elder Life Program (HELP), a multicomponent targeted intervention to prevent delirium in hospitalized older patients: efficacy and cost-effectiveness in Dutch health care

**DOI:** 10.1186/1471-2318-13-78

**Published:** 2013-07-23

**Authors:** Marije J Strijbos, Bas Steunenberg, Roos C van der Mast, Sharon K Inouye, Marieke J Schuurmans

**Affiliations:** 1 Department of Rehabilitation, Nursing Science and Sports Medicine, University Medical Center Utrecht, Heidelberglaan 100, Utrecht 3584 CX, The Netherlands; 2 Julius Center for Health Sciences and Primary Care, University Medical Center Utrecht, Universiteitsweg 100, Utrecht 3584 CG, The Netherlands; 3 Department of Psychiatry, Leiden University Medical Center, Postbus 9600, Leiden 2300 RC, The Netherlands; 4 Department of Medicine, Beth Israel Deaconess Medical Center, Harvard Medical School, and the Institute for Aging Research, Hebrew Senior Life, 1200 Center Street, Boston, MA 02131, USA

**Keywords:** Delirium, Prevention, Design and methods, Hospital care organization, Older people, The Netherlands, Hospital elder life program

## Abstract

**Background:**

The Hospital Elder Life Program (HELP) has been shown to be highly efficient and (cost-)effective in reducing delirium incidence in the USA. HELP provides multicomponent protocols targeted at specific risk factors for delirium and introduces a different view on care organization, with trained volunteers playing a pivotal role. The primary aim of this study is the quantification of the (cost-)effectiveness of HELP in the Dutch health care system. The second aim is to investigate the experiences of patients, families, professionals and trained volunteers participating in HELP.

**Methods/Design:**

A multiple baseline approach (also known as a stepped-wedge design) will be used to evaluate the (cost-) effectiveness of HELP in a cluster randomized controlled study. All patients aged 70 years and older who are at risk for delirium and are admitted to cardiology, internal medicine, geriatrics, orthopedics and surgery at two participating community hospitals will be included. These eight units are implementing the intervention in a successive order that will be determined at random. The incidence of delirium, the primary outcome, will be measured with the Confusion Assessment Method (CAM). Secondary outcomes include the duration and severity of delirium, quality of life, length of stay and the use of care services up to three months after hospital discharge. The experiences of patients, families, professionals and volunteers will be investigated using a qualitative design based on the grounded theory approach. Professionals and volunteers will be invited to participate in focus group interviews. Additionally, a random sample of ten patients and their families from each hospital unit will be interviewed at home after discharge.

**Discussion:**

We hypothesize that HELP will reduce delirium incidence during hospital admission and decrease the duration and severity of delirium and length of hospital stays among these older patients, which will lead to reduced health care costs. The results of this study may fundamentally change our views on care organization for older patients at risk for delirium. The stepped-wedge design was chosen for ethical, practical and statistical reasons. The study results will be generalizable to the Dutch hospital care system, and the proven cost-effectiveness of HELP will encourage the spread and implementation of this program.

**Trial Registration:**

Netherlands Trial register: NTR3842

## Background

In the next 30 years, the proportion of people 65 years and older is expected to increase to account for 25% of the general Dutch population, compared to 15% in 2008 [1]. Older people are at a higher risk for hospitalization. Of every 10,000 people 65 years and older, 3,131 older people were admitted to the hospital in 1995 and 5,521 in 2010 [2]. In 2005, 25 % of the admitted patients in hospitals were 70 years old or older, for totaling 400,000 people [3]. The risk for delirium is significantly associated with age, and in 2005, the Dutch Health Care Inspectorate (IGZ) indicated that 40,000 to 160,000 hospitalized patients 70 years and older suffer from delirium annually in the Netherlands [3].

Delirium is characterized by an acute disruption of attention and cognition [4] and is the main cause of confusion in the general hospital [5]. The DMS-IV criteria for delirium are as follows: a disturbance of consciousness with a reduced ability to focus, sustain, or shift attention; a change in cognition (memory, language, or orientation) or the development of a perceptual disturbance not related to dementia; a quick onset that fluctuates during the day; and an underlying physiological problem or medication as the cause [4].

Patients with delirium are at an increased risk for complications and mortality during a hospital stay. Delirium can further result in adverse events after hospitalization, including lasting functional limitations, persistent cognitive decline, loss of quality of life for the patient and family, re-hospitalization and nursing home admission [6-8]. Unfortunately, nurses and physicians often do not recognize the presence of delirium [5]. Once delirium is diagnosed, standard delirium treatment consists of diagnosis and treatment of the underlying medical condition. Furthermore, management of delirium consists of environmental interventions addressing orientation, day structure, safety and, if necessary, the prescription of (antipsychotic) drugs [5].

Preventing delirium is preferred over treating delirium, and attention should therefore focus on (multi-faceted) prevention interventions. Recently, a meta-analysis of five studies of 1,491 older patients concluded that the perioperative use of antipsychotics may reduce the overall risk of postoperative delirium among older people [9], but the results to date have been inconsistent [10] and some reveal only decreased delirium duration or severity, with either no impact or adverse impact on clinical outcomes [11].

Relatively little high quality research on delirium prevention (not including postoperative treatment) has been published [12], and an approach targeting several risk factors for delirium appears to be the best strategy to prevent delirium [13,14]. Currently, there is increasing evidence that delirium can be prevented through non-pharmacological interventions [10,14,15].

### Aims

In this project, the primary aim is to investigate the (cost-)effectiveness of the Hospital Elder Life Program (HELP) in the Dutch hospital care system; this program has been shown to be effective in the USA for the prevention of delirium in older adults during hospital admission to both internal medicine and surgery departments. HELP has been shown to be effective in reducing the incidence, duration and severity of delirium [15] and decreasing health care costs [16-19]. A second aim is to investigate the experiences of patients, families and volunteers involved in HELP because in the USA, HELP also enhanced patient and family satisfaction with hospital care and improved the quality of care [16]. The introduction of HELP requires a fundamentally different view on the care organization and a change in the care process, including the introduction of methods for early recognition of delirium symptoms combined with care plans, the introduction of an Elderly Care Nurse Practitioner position and the introduction of trained volunteers to provide additional care and psychosocial support.

Although HELP has been disseminated at a number of USA and internationally [20,21], only recently has the first implementation of an adapted HELP program in a European hospital (Spain) been described [22]. The Dutch hospital care system and the patient population are different from the system and patients in the USA. Therefore, it is not possible to extrapolate earlier findings on the effects of HELP to the Dutch system. The HELP materials and protocols will be translated and adapted where necessary in close collaboration with the developer of the HELP program, Dr. Sharon Inouye.

## Methods/Design

### Design

A multiple baseline approach (also known as a stepped-wedge design) [23] will be used to evaluate the efficacy and (cost-) effectiveness of the introduction of HELP within the Dutch health care system (Additional file 1: Table S1).

Over a period of 18 months, eight hospital units of the Hospital Gelderse Vallei in Ede and the Diakonessenhuis in Utrecht and Zeist in the Netherlands will successively receive the intervention; new units will start every three months.

To investigate the experiences of patients, families, professionals and trained volunteers involved with the HELP program, a qualitative design study based on the grounded theory approach [24,25] will be conducted.

### Study population

Eligible participants are patients ages 70 years and older who are at risk for delirium and are admitted to the cardiology, internal medicine, geriatrics, orthopedics and surgery units of the two hospitals. The Diakonessenhuis is situated in two locations (one in a university city and one in a smaller town nearby), while the Hospital Gelderse Vallei is located in a more rural area.

Eligible patients will be approached by the nurse on call for participation within 24-hours after hospital admission. Inclusion criteria are being 70 years and older, the absence of delirium at hospital admission and being considered at risk for delirium.

To assess whether a patient is at risk for delirium, we use the three questions of the Hospital Safety Program launched in the Netherlands in 2009 that are part of obligatory hospital care. A review of literature was done before choosing these questions [26]. The nurse will assess the risk for delirium in patients 70 years and older with the following questions: “Do you have memory problems?”; “During the past 24-hours, did you need assistance with your daily self-care?”; and “Were you confused during earlier hospital admissions or illnesses?”. When at least one of these questions is answered positively, there is an increased risk of delirium.

Exclusion criteria include a life threatening situation, being in a palliative phase at admission, an expected hospital stay of 24 hours or less, being legally incapable of participating, unable to communicate verbally, or a second hospital admission during the study period. If the patients are transferred to a participating unit, they are treated as a newly admitted patient; if the patients are transferred from a participating unit to a nonparticipating unit, they are excluded from the study.

### Sample size

The sample size calculation was based on the primary endpoint incidence of delirium, as diagnosed by the Confusion Assessment Method (CAM) [27]. According to the literature, 10-40% of older hospitalized patients develop delirium [28-30]. HELP has been shown to reduce the absolute rate of delirium by 14.4%, which represented a relative risk reduction of 35.3% [17]. Thus, the difference in delirium incidence between the HELP-intervention group and the care-as-usual group was conservatively estimated to be at least 10%. To demonstrate this difference, using a two-sided test with an alpha of 0.05 and a power of 0.90, two groups of 470 patients are required, indicating a study population of 940 patients. Estimating that 15% of patients will not be willing to participate, the total number of patients to be included was estimated to be 1081 patients.

A total of 1648 patients ages 70 and older are hospitalized annually in the Diakonessenhuis. In the Hospital Gelderse Vallei, a total of 1393 patients aged 70 years and older are hospitalized annually. These hospitalizations result in 3,041 patients admitted per year, of whom at least two-thirds are expected to be at risk for delirium. This expectation is based on figures from hospitals that have already implemented the three question delirium risk assessment. This calculation should be sufficient for determining the total number of participants that are needed (n = 1,081) for the proposed study.

### The intervention

The Hospital Elder Life Program (HELP) is an innovative program for the prevention of delirium in hospitalized older people. The strengths of the program include the targeted nature of the interventions, early intervention focusing on prevention, well-trained staff dedicated to the program, standardized intervention protocols, tracking of adherence to all protocols, and built-in quality assurance procedures [16]. The four primary goals of the program to prevent delirium are the following: to maintain cognitive and physical functioning of high risk older adults throughout hospitalization, to maximize independence at discharge, to assist with the transition from hospital to home and to prevent unplanned hospital readmissions [31]. The program has four components:

1) ***Protocols targeting risk factors***

The program provides standardized protocols targeted toward six important delirium risk factors. These factors were selected on the basis of evidence of their association with the risk of delirium and because they were amenable to intervention. These risk factors include cognitive impairment, sleep deprivation, immobility, visual impairment, hearing impairment, and dehydration.

2) ***Elderly care nurse practitioner***

The Elderly Care Nurse Practitioner, who will be introduced as part of the program, plays a central role in HELP. The nurse practitioner will provide a baseline geriatric assessment upon admission and during the project, will develop and implement interventions in collaboration with the nursing staff and other disciplines and will include the patients in the process. This nurse will also provide educational programs and bedside teaching for nursing staff and will coordinate interdisciplinary rounds. The nurse practitioner will receive extensive training concerning assessment of the questionnaires and measurements and how to obtain informed consent. The nurse practitioner will work in close collaboration with a geriatrician.

3) ***Trained volunteers***

Trained volunteers are a unique feature of HELP and are innovative in Dutch hospital care. The coordination of all volunteer activities in HELP will be conducted by a specially trained coordinator and the nurse practitioner. The trained volunteers play a crucial, central role in HELP by conducting the interventions directly at the bedside. The volunteers will stimulate patients to eat, drink and walk; read newspapers with the patients, and participate in (word) games and other activities with the patients. The volunteer training consists of classroom instruction that includes didactic training, small group demonstration, role playing and case discussions. Volunteers will communicate with each other, the nurse practitioner and the nurses during each volunteer shift. Volunteers will be additionally coached and trained quarterly with educational sessions and discussion groups.

4) ***Patient-friendly***

HELP is unique in its approach of being patient-friendly by being directed at the wishes and needs of the older patients and in guaranteeing additional psychosocial support. Patients will receive personalized interventions that match their changing needs throughout the course of hospitalization. Enrolled patients will be regularly reassessed by the HELP staff, and nurses and supervised volunteers will administer interventions that are reassigned daily and tracked for adherence.

All HELP protocols, training manuals and procedures are available for on-site registered hospital units wishing to start a HELP site [31]. For this study, all of the materials will be translated and adapted, if necessary, to Dutch in close collaboration with the developer of HELP, Dr. Sharon Inouye. Subtle changes will be made to the program to fit the Dutch hospital care system. In the original HELP manuals, an Elder Life Specialist and an Elder Life Nurse Specialist were involved. For this project, the titles nurse practitioner and volunteer coordinator are used. Table 1[32] provides an comparison of the original HELP protocols and HELP adapted to the Dutch situation.

**Table 1:** Comparison US and Dutch HELP

	**Original HELP protocols**	**HELP in this study**
Goals	Maintain physical and cognitive functioning, maximize independence at discharge, assist with the transition from hospital to home, prevent unplanned readmission.	No adaptation
Screening	ELS within 48 hours	NP within 36 hours
Inclusion criteria	70 years and over, at least one risk factor for delirium present	70 years and over at risk for delirium according to Dutch Safety Management Program
Exclusion criteria	intubation or respiratory isolation, aphasia, terminally ill, severe dementia, respiratory isolation, expected discharge within 48 hours after admission.	Same exclusion criteria except; exclusion when discharge is expected within 24 hours after admission. An added exclusion criterion; a second admission to a participating unit.
Protocols	Daily visitor program, feeding assistance program, early mobilization program, therapeutic activities program.	No adaptation
Volunteer shifts	Ranging from one to three times daily across protocols	Two times daily, one in the morning, one in the evening
HELP staff	Program director: oversees and supervises the entire program within a hospital.	Project leader :oversees all aspects the project within a hospital.
	Elder Life Specialist: responsible for day-to-day operations of the program, patient screening and coordination of the volunteers.	Volunteer coordinator: screens volunteers, makes sure volunteers attend the training, coordinates and provides support volunteers.
		Nurse Practitioners: screen patients, complete instruction forms for volunteers.
	Elder Life Nurse Specialist: clinical assessment and intervention skills, develops and implements practical strategies to prevent cognitive and functional decline, provides education to nursing staff, liaison with other health care specialties.	Nurse Practitioners: complete the measurements on delirium, quality of life, and cognitive function. They are in close contact with the nurses and instruct them when necessary.
	Geriatrician: provides geriatric assessment and consultation upon request, education to physicians, liaison with hospital medical staff	No adaptations
Staff nurses	ELS and ELNS are in contact with the staff nurses.	NP’s and volunteers are in contact with the staff nurses. The volunteers communicate with the staff nurses on patient level at the start and end of their shift.
Outcomes	Advised: brief cognitive screening test, such as SPMSQ, Activities of Daily Living scores, vital status, length of hospital stay, discharge destination, use of home services, hospital costs.	Incidence, duration and severity of delirium, 6-CIT, Activities of Daily Living Scores, diagnosis, length of stay, care consumption after discharge, health care costs, quality of life.

SPMSQ = Short Portable Mental Status Questionnaire [32].

### The implementation

Three months prior to the first hospital unit starting with HELP, all participating units will receive presentations on the content of the study and the measurements. The purpose of this period is to draw attention to the measurements that nursing should use to assess a patient’s risk for delirium. Baseline data will be collected during this time.

As described, every three months, one hospital unit in both hospitals will start the intervention. The four hospital units in both hospitals are comparable. The units that admit the most older patients were chosen. The staff will be made aware of the study’s time schedule and will know when they are in the control or intervention period. Before the unit starts with the intervention, a team of HELP volunteers will be assembled and will receive a two day training program that was specially developed for HELP. The researcher and local project leader will work closely with the volunteer coordinator, the trainers and the head of the hospital unit. The researcher will give regular presentations for the nurses of the hospital unit and will keep the hospitals informed with regular newsletters and messages on the hospitals’ webpages. The doctors, physical therapists and other professionals in the hospital unit will be informed by the project leader.

Each hospital has created a group of experts that gathers approximately every two months to discuss the HELP plans and its progress. This group can consist of the head and nurses of the participating hospital units, a doctor, the volunteer coordinator and the project leader.

### Study procedure

**Figure 1:**
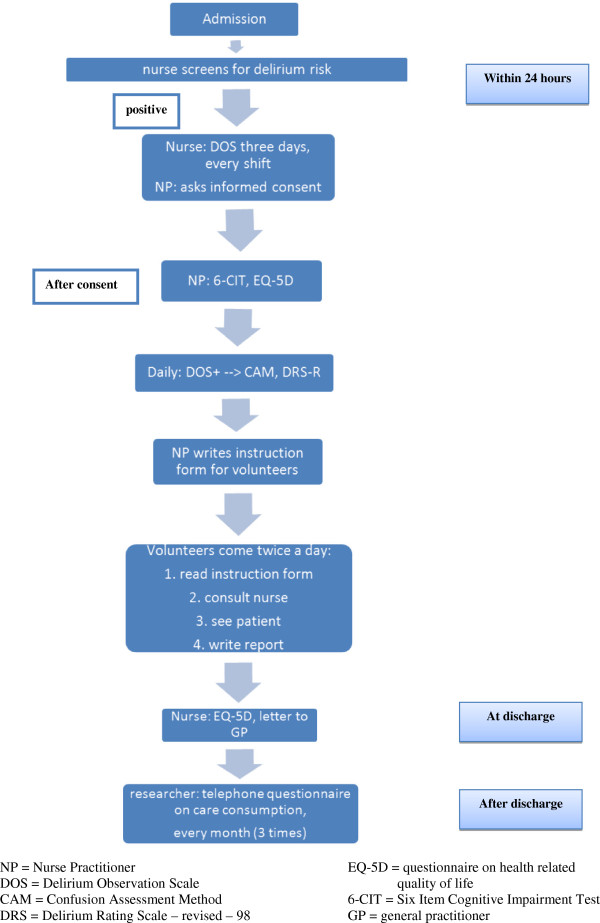
Measurements during the intervention period.

Figure 1 provides an overview of the measurements and questionnaires used during the intervention period of the study. The measurements are the same in the control period, except for the presence of the HELP volunteers. Eligible patients will be approached by the nurse on call for participation within 24-hours after hospital admission, and the nurse practitioner will measure delirium presence, severity and duration as described in the figures. Most of the questionnaires are part of the current hospital care for older people [26].

### Ethical considerations

The HELP study has been approved by the Medical Ethics Board of the University Medical Center of Utrecht. Both the Hospital Gelderse Vallei in Ede and Diakonessenhuis in Utrecht and Zeist have reviewed the study protocol. The board concluded that the study was feasible. Informed consent will be obtained, and patients can always decide not to participate. The study is registered in the Netherlands Trial Register (NTR3842).

### Measurements

#### Six-item cognitive impairment test

The Six-Item Cognitive Impairment Test (6-CIT) is a brief cognitive test that correlates well with the Mini-Mental State Examination (MMSE). The use of this test as a screening instrument for older people in hospital care has been studied and compared to the MMSE [32]. The sensitivity and specificity of the 6-CIT were 0.90 and 0.96, respectively, and the positive and negative predictive values were 0.83 and 0.98, respectively. The 6-CIT was chosen over the MMSE because of its short time to administer (2.5 minutes versus 5.8 minutes for the MMSE) and because the 6-CIT does not require good language skills, reading or writing and is not sensitive to educational level [33].

The 6-CIT will be administered by the nurse practitioner within 36 hours after admission, will be used to gather information on cognitive functioning, and can help inform the decision to consider a patient for participation.

### Delirium outcomes

The incidence of delirium will be diagnosed with the Confusion Assessment Method (CAM) [27], whereas the duration (number of days) and severity of the delirium will be assessed using the Delirium Rating Scale (DRS-R) [34]. In patients who fulfill the selection criteria, ward nurses will rate delirious symptoms daily using the Delirium Observation Scale (DOS) [35] at the end of each shift until discharge. This approach is already part of standard clinical practice and the VMS. When the patients receive a positive score for the DOS, the CAM will be administered by the nurse practitioner or geriatrician. In patients diagnosed with delirium according to the CAM, the Delirium Rating Scale (DRS-R) [34] will be administered daily by the nurse practitioner.

#### The confusion assessment method (CAM)

The CAM, a 4-item diagnostic instrument, was designed as a screening tool for people who are at high risk of delirium [27] and is seen as the gold standard in most delirium studies [30].

The CAM assesses the presence of four delirium features: acute onset and fluctuating course, inattention and distractibility, disorganized thinking and illogical or unclear ideas, and an alteration in consciousness. A review concluded that the CAM has become widely used, mainly because of its ease of use and accuracy [36,37]. The overall sensitivity appeared to be 94% and the specificity 89%; interrater reliability was high (0.70-1.00). In addition, the CAM has been translated into over 15 languages, including Dutch [36].

#### The delirium observation scale (DOS)

The DOS was developed to facilitate early recognition of delirium, based on nurses' observations during regular care; this scale uses DSM-IV criteria for delirium, a literature review, and clinical experiences [35]. This measure consists of 13 items that are rated on a 3-point scale based on the frequency of occurrence of the behavioral change, including never (0 points), sometimes–always (1) or do not know (−). A total score of three or higher is indicative of being delirious [35]. The DOS scale was determined to be valid and showed high internal consistency. Predictive validity against the Diagnostic and Statistical Manual-IV diagnosis of delirium made by a geriatrician was good [30]. The DOS had reported sensitivities of 0.89 and 0.94 and specificities of 0.77 and 0.88 [38].

#### The delirium rating scale (DRS-R)

The Delirium Rating Scale was developed in 1988 and revised in 1998 [34]. This scale was originally designed to be used by psychiatrists but can also be used by other physicians, nurses and psychologists with appropriate training. The DRS-R-98 is a 16-item clinician-rated instrument with 13 severity items and 3 diagnostic items. The maximum total score is 46 points, and the maximum severity score is 39 points [34]. To rate the items on the DRS, all sources that are available are used (e.g., nurses, family and medical files) [34]. Interrater reliability (ICC = 0.98) and internal consistency were very high, and the sensitivity and specificity were 92% and 93%, respectively [34].

### Quality of life outcomes

Health related quality of life (general quality of life, functional wellbeing and emotional wellbeing) will be measured with the EQ-5D [39] at baseline (admission to the hospital) and at discharge from the hospital. The EQ-5D is a multidimensional measurement of health consisting of the EQ-5D descriptive system and the EQ VAS. The EQ VAS records the respondents self-rated health status on a vertical graduated (0–100) visual analogue scale. The EQ-5D descriptive system comprises 5 dimensions of health (mobility, self-care, usual activities, pain/discomfort and anxiety/depression).

Each dimension comprises three levels (no problems, some/moderate problems or extreme problems). The EQ-5D may be used to calculate quality adjusted life years (QALYs).

Test-retest reliability has been shown to be good, and the test is easy to use to measure both the presence of clinical symptoms and changes in these symptoms over time [39].

### Qualitative outcomes

The quality of care will be examined by means of focus groups. The quality of the care “process” experienced by the patients will be studied at each of the participating units during the period of data collection. Focus group meetings will be held with the multidisciplinary HELP-team. These focus groups will be led by a qualitative researcher with the assistance of the HELP-trained professionals and research-project members. The HELP program, care pathways, and the cooperation within the multidisciplinary team are topics that will be discussed by these focus groups. Next, qualitative interviews with the patients and their family members will be conducted. The objective of these interviews is to collect information on the experiences and opinions about the HELP program from both the patients and their next of kin.

Furthermore, the HELP volunteers will participate in a so called focus group to evaluate their role and experiences; each unit will have one group that participate every month during the first half year and then every two months during the period of data collection.

### Cost-effectiveness outcomes

The primary effect parameter of the economic outcome evaluation will be the number of prevented cases of delirium. The costs of the HELP program will be calculated as well as the costs of care consumption of both patients who received HELP and patients who did not both during and after their hospital stay. After discharge, the patients will be contacted three times by telephone during a period of three months for assessment of their care consumption. During this telephone call, patients will be asked eight short questions addressing the following topics: possible hospital (re)admissions, nursing home or rehabilitation center admissions, home care, domestic help, informal care, visits from the general physician and psychological care. If the patients are not able to participate, a close family member will be asked for information on the care that the patients have received after discharge. Differences in total cost between the intervention and control groups will be compared.

### Statistical analysis

The aim of the main analysis is to compare the incidence, severity, and duration of delirium in pre- and post-HELP patient groups. To correct for the clustering of patients within wards and for baseline characteristics, multi-level analysis using the R statistical package will be used to evaluate differences in treatment outcomes between the two groups [40]. Estimates will be reported with 95% confidence intervals. The analysis will be conducted according to the intention-to-treat principle.

Missing data will be corrected using multiple imputation.

### Qualitative analyses

The aim of the qualitative research question is to incorporate viewpoints to be included in future HELP-training protocols and HELP-manuals for Dutch institutions that want to implement HELP. This information will enhance the chance of a successful implementation.

The qualitative study will use a grounded theory approach to analyze the experiences of patients and HELP-team members. Grounded research combines two data analysis processes; all data are coded and systematically analyzed to verify or prove a proposition [25]. The researcher does not necessarily engage in coding data but merely inspects the data for categories, makes memos and develops ideas [25]. Emerging themes during the process of data collection lead to implementation of these themes in following interviews. To examine the content of the interviews, an encoding scheme using a systematic and iterative method will be employed. The goal of this process is to elicit the meaning of an experience from the viewpoint of those who have had that experience. Data will be transcribed and analyzed using computer assisted qualitative data analysis software, Nvivo 9 (QSR International).

### Costs-evaluation

Differences in total mean costs between groups will be related to the differences in the number of cases of delirium and of the QALYs gained after three months. QALYs will be calculated by multiplying the utility of a health situation by the time spent in these health situations. The incremental cost effectiveness ratio (ICER) will be calculated and graphically presented as a scatter of bootstrapped ICERs on the cost-effectiveness plane and as an acceptability curve for a series of willingness to pay ceilings. Sensitivity analyses will be conducted that will focus on the most salient cost-drivers.

## Discussion

This is the first study to implement and study efficacy and cost-effectiveness of the Hospital Elder Life Program (HELP) in the Netherlands. HELP has proven (cost-)effective in the US and Canada, and for that reason, the project group decided to stay as close to the HELP protocols as possible when implementing HELP in the Dutch hospital care system. Necessary adjustments, including a more involved role for the nurses and specific delirium screening methods, were discussed with and approved by Dr. Inouye, the developer of HELP.

A controlled clinical trial with prospective, individual matching to compare patients admitted to one intervention and two usual-care (control) units was used in the original HELP study [13]. Randomization on the level of patients was not possible due to logistic reasons. This design demands comparable units, of which one can be prepared as an intervention unit and one as a control unit. However, in Dutch hospitals, only one unit per medical specialty is available. The stepped-wedge design is a specific cluster randomized controlled study design that is useful for the evaluation of patient safety interventions, such as HELP [41,42]. Moreover, the stepped-wedge design has statistical advantages. Hospital units act as their own control and hence provide data points for both the control and intervention units [41,42]. This feature of the stepped-wedge design reduces the risk of bias, which may be the most important issue in non-randomized studies. HELP will be implemented in two hospitals in two different cities, one of which is located in a rural area. The hospitals are similar in size, and the chosen units are comparable; these factors increase the generalizability of the results of this study to the Dutch hospital care system. The hospitals involved in this project are motivated to implement HELP and admit a relatively high number of older patients as a result of the demographics in their regions.

In the Netherlands, screening for delirium in older people has been a quality of care indicator of the Dutch Health Care Inspectorate (IGZ) since January 2010. As a result, hospitals are required to report delirium incidence and to develop policies on delirium prevention [26]. Our data collection closely aligns with this quality measure and strengthens the hospitals’ desire to participate. Given the stepped-wedge design and the fact that measurements are part of regular care, the Nurse Practitioners who are responsible for measuring the primary outcome know which patients receive the intervention. The lack of blinding is preempted by written protocols for screening, and diagnosis as well as the highly standardizes intervention. Moreover units are their own controls and at the same time their data can be compared to data of a comparable unit in the other hospital. To overcome the lack of blinding and ensure reliability of the primary outcome much emphasis will be given to training of the Nurse Practitioners. In addition, CAM interrater reliability between the Nurse Practitioners and between the Nurse Practitioner and a geriatrician will be tested regularly during the study. Finally delirium incidence can be compared with historical data of the participating wards using the procedure described by Rubin et al. in 2006.

Although delirium screening is part of regular care, the protocols are not always followed; this study may improve the percentage of screening for delirium. Thus, an increase in the number of patients with delirium risk is expected, most likely resulting in more delirium diagnoses. Three months prior to the start of the study, the researchers will pay close attention to the measurements that should be part of regular care to achieve the same starting point for all units. This coordinated effort will result in reliable baseline data on delirium risk and delirium diagnosis in all participating hospital units. If HELP proves to be effective, the chance of implementation of the protocol is largely due to the alignment of the aims with screening regulations and developments in health care standards.

Much attention will be paid to the recruitment, selection, training and coaching of volunteers. These individuals will receive a two day training and support from volunteer coordinators; a meeting will be organized every three months to allow the volunteers to share and evaluate their experiences. Before and after their activities, volunteers will also need to exchange information with nurses, which involves a change in the nurses’ work. The accessibility of the nurses will require attention from the project group.

A concern is the participation of an adequate number of older patients. The literature suggests that in older people, the refusal rate is higher than in younger populations. Several possible causes for this factor have been suggested. One reason described is that a lengthy and complicated patient letter with risks and benefits can be difficult to read and understand for frail older people [43]. The Medical Ethical Board considered this a low risk study; however, the Board has required that informed consent be obtained using a detailed and lengthy information letter, which may suggest that invasive research activities rather than additional care are being offered. It is therefore expected that fewer patients will be willing to sign consent.

HELP requires a fundamentally different view on the care organization and a change in the care process, including the introduction of methods of early recognition combined with care plans and the introduction of an Elderly Care Nurse Practitioner and trained volunteers to provide additional psychosocial support. Results of one cost-effectiveness study [17] showed that each patient who was prevented from becoming delirious saved the hospital $2,181. Although HELP has been implemented at a number of U.S., Canadian, Australian, and Taiwanese sites, only recently has the first implementation of a HELP-like program in a European hospital (Spain) been published [22]. The Dutch hospital care system and patient population are different than those of the USA. These differences have our utmost attention; in cooperation with the developers and users, we will try to implement HELP as closely as possible to the original protocols.

### Conclusion

This research project aims to quantify the efficacy and (cost-) effectiveness of the Hospital Elder Life Program (HELP) in the Dutch health care system. The project will be extended with a qualitative study to describe and understand the experiences of patients, families, professionals and trained volunteers. HELP is unique in its approach by being patient-friendly, oriented to the wishes and needs of the older patients and guaranteeing additional psychosocial support. If HELP is proven (cost-) effective in this study, we will initiate knowledge transfer and implementation on a national level.

## Competing interests

The authors declare that they have no competing interests.

## Authors’ contributions

MS drafted this manuscript. MS, BS, RM and SI were actively involved in writing the manuscript. All authors read and approved the final manuscript.

## Pre-publication history

The pre-publication history for this paper can be accessed here:

http://www.biomedcentral.com/1471-2318/13/78/prepub

## Supplementary Material

Additional file 1: Table S1 Stepped-wedge design. Click here for file
