# Supplementary material for: Design and methods of the Hospital Elder Life Program (HELP), a multicomponent targeted intervention to prevent delirium in hospitalized older patients: efficacy and cost-effectiveness in Dutch health care
Source: BMC Geriatr. 2013 Jul 23;13:78. doi: 10.1186/1471-2318-13-78 (PMC3724594; doi:10.1186/1471-2318-13-78)
Supplement: Additional file 1: Table S1 — Stepped-wedge design. [file 1471-2318-13-78-S1.docx]

**Additional file 1: Table S1: stepped-wedge design**

| Hospital/Unit | Time Periods – Study Period Introducing HELP to participating units | | | | | |
| --- | --- | --- | --- | --- | --- | --- |
|  | 1 – 3 months | 4 – 6 months | 7 – 9 months | 10 – 12 months | 13 – 15 months | 16 – 18 months |
| Gelderse Vallei Unit* 1 |  |  |  |  |  |  |
| Unit 2 |  |  |  |  |  |  |
| Unit 3 |  |  |  |  |  |  |
| Unit 4 |  |  |  |  |  |  |
|  |  |  |  |  |  |  |
| Diakonessenhuis Unit 1 |  |  |  |  |  |  |
| Unit 2 |  |  |  |  |  |  |
| Unit 3 |  |  |  |  |  |  |
| Unit 4 |  |  |  |  |  |  |

*Unit = Hospital unit, random allocation

Blank cells represent control periods

Shaded cells represent intervention periods

Each cell represent a data collection point
